# The relationship between students’ personality traits, attention state, and use of regulatory strategies during emergent distance learning

**DOI:** 10.1186/s40359-025-02451-3

**Published:** 2025-02-13

**Authors:** Mona Emara, Susanne Schwab, Ghaleb Alnahdi, Cornelia Gerdenitsch

**Affiliations:** 1 Department of Educational Psychology, Faculty of Education, Damanhour University, Damanhour, Egypt; 2 STEM Department, Linz School of Education, Johannes Kepler University, Linz, Austria; 3 University of Vienna (Centre for Teacher Education), Porzellangasse 4, Vienna, 1090 Austria; 4 North-West University, Research Focus Area Optentia, Vanderbijlpark, South Africa; 5 Department of Special Education, College of Education, Prince Sattam Bin Abdulaziz University, Al-Kharj, Saudi Arabia; 6 AIT Austrian Institute of Technology GmbH, Vienna, Austria

**Keywords:** Distance learning, Attention distraction, Personality traits, Social Media

## Abstract

**Background:**

Attention issues are increasingly prevalent among students in higher education. While existing research has primarily focused on external distractions and their relationship with personality traits, internal distractions remain underexplored, particularly in the context of specific learning scenarios like distance education. This study addresses this gap by investigating the correlations between personality traits, attention distraction, and attentional regulation in the context of distance learning.

**Method:**

This study adopted a combined person- and variable-oriented approach to examine the extent to which students’ personalities relate to attentional state and regulation strategies during emergent distance learning under COVID-19 lockdown conditions. 400 higher education students completed an online survey for this cross-sectional study.

**Results:**

The integrated approaches revealed three distinct groups: (1) the “Self-Attention Regulated” group, characterized by the lowest attention problems, the highest use of attention regulation strategies, and higher levels of conscientiousness and openness; (2) the “Hanging-On” group, marked by high attention discontinuity, moderate attention regulation strategies, and average levels across all personality traits; and (3) the “Social Media-Distracted” group, exhibiting the highest levels of distraction caused by social media and higher levels of neuroticism. Older students, who tended to belong to the self-attention-regulated group, spent considerably more time studying online than younger students, who were more likely to be social media-distracted.

**Conclusion:**

This study enhances our understanding of attention regulation in distance learning by identifying personality traits associated with students at risk for distracted attention issues. The results could help universities to design and implement effective distance learning programs that cater to a wide range of student personalities.

## Introduction

In response to the lockdowns arising from the COVID-19 pandemic, digital devices have been widely used to enable distance education [1]. Some students may benefit from distance learning, but others may be at risk because they are easily distracted and have limited attention [2, 3].

To understand why certain students behave differently, the literature suggests that at-risk groups may struggle to develop attention regulation skills [4], which include the ability to voluntarily focus on one task at a time and control potential distractions [5, 6]. Failure to regulate attention can lead to concurrent engagement in multiple media applications that are irrelevant to learning. This phenomenon is referred to as media multitasking and is widely prevalent among university students [7].

Besides cognitive abilities, evidence suggests that multitasking and attention proficiency also depend on personality traits. Characteristics such as polychronicity—the preference for engaging in multiple tasks concurrently—and traits identified within the Big Five personality model [8], including extraversion, conscientiousness, agreeableness, openness to experience, and emotional stability, have been implicated in varying capacities for managing distractions [9].

Despite the recognition of these individual differences, there remains a gap in synthesizing how cognitive abilities and personality traits interact with the propensity for internet and social media related distractions among higher education students engaged in distance learning. Moreover, the limited empirical evidence on the connection between personality and attention distraction has predominantly utilized a variable-oriented approach —focusing on group-level associations. For example, neurotic introverts perform worse under distraction and show greater attentional deficits than stable extroverts [10, 11]. While these findings are revealing, they offer limited insight into how personality, attention problems, and regulatory strategies interact.

Addressing this gap, the present study blends variable-oriented and person-centered approaches. While the variable-oriented approach illuminates patterns among variables at the group level, the person-centered approach [12, 13] highlights the unique interplay of factors that define an individual’s performance. By weaving these perspectives together, our analysis offers a nuanced view of students’ attention profiles and regulatory strategies, illustrating how various traits and behaviors intersect in the distance learning environment. This insight will support the development of more effective and practical distance courses.

### Distance learning, distractions due to social media and regulation skills

Distance learning effectiveness largely depends on students’ ability to self-regulate their attention [14–16]. Attention is a central cognitive process that governs the efficient selection and allocation of mental resources required for information processing. Models of attentional processing, such as Kahneman’s Capacity Model [17], highlight how attentional resources are finite and influenced by task demands. In the context of distance learning, distractions—both internal (e.g., mind-wandering) and external (e.g., social media)—can impede the ability to encode, retrieve, and apply knowledge effectively. Working memory plays a critical role in attentional control. According to Baddeley’s Working Memory Model [18, 19], the central executive system regulates attentional processes and coordinates information from sensory inputs and long-term memory. This model emphasizes that cognitive overload or distractions can impair the working memory's capacity, negatively impacting learning outcomes.

Recent studies highlight social media as a major distraction, particularly during emergent situations like the COVID-19 pandemic, affecting young adults and students [20, 21]. In such situations, the allure of social media notifications and updates can interrupt students’ primary attention, compromising learning objectives [4, 22]. Wu [4] applied cluster analysis to examine the impact of digital and social media use on students' attention states and regulation in online learners, resulting in a meta-attention framework with two components: knowledge of attention (awareness of one’s attention state and ability to identify distractions) and attention regulation (the strategies employed to maintain concentration). The cluster analysis revealed distinct profiles, with group differences in attention challenges, regulatory strategies, online search behaviors, and time spent on the Internet and social media.

Although numerous studies document the prevalence of distractions and the necessity for attention regulation in distance learning, the underlying factors that predispose certain students to be more easily distracted remain less understood [4, 23, 24]. Research suggests that both attention and higher-order meta-attention processes are critical for initiating and sustaining cognitive engagement [4, 25], yet students exhibit notable individual differences in sustaining concentration and managing distractions in distance learning contexts.

### Personality traits, meta-attention, and distance learning

The Big Five Model of personality [8] categorizes traits into extraversion, agreeableness, conscientiousness, neuroticism, and openness, providing a robust framework for understanding individual differences. Prior research has extensively explored the relationship between these personality traits and social media usage [26, 27], and its connection to attention distraction [28, 29]. For instance, studies have reported conscientiousness is negatively associated with social media use [30], while openness and agreeableness show positive relationships [21, 27]. Neuroticism yields mixed findings, with studies reporting either no link or a positive association [26]. Extraversion consistently correlates positively with social media use [27].

From an information-processing perspective [31], distractions, such as social media notifications, can disrupt the flow of information from sensory registers to working memory and long-term storage. Personality traits may influence how learners manage attention during this process. For example, Eysenck et al. [32] suggest that anxiety, often linked to neuroticism, impairs attentional control and heightens distractibility.

Furthermore, studies suggest that the relationship between personality traits and social media distractions during multitasking may vary across individuals [33, 34]. Media multitasking, defined as switching attention between activities, can be driven internally (e.g., boredom) or externally (e.g., social media notifications) [35, 36]. While media multitasking-related distractions are prevalent among distance learners in higher education, individual differences significantly influence these behaviors [4, 23, 24]. For example, Ma et al. [37] demonstrated that personality traits like openness to experience and conscientiousness moderate the cognitive effects of multitasking. Conscientious individuals showed better focus and task management, reducing social media-related distractions, whereas those with higher openness engaged more positively in multitasking scenarios [37].

The current study differs from other educational efforts in that we combined person- and variable-oriented approaches to explore how different students’ personalities impact their attentional states and regulation strategies when confronted with distance learning. More specifically, we focused on investigating the relationship between the personalities of higher education students and their attention distraction due to social media and their use of attention-regulation strategies during the COVID-19 lockdown. It specifically aimed to answer the following research questions:

*Are personality traits and the growing use of distance learning affecting how higher education students react to internet and social media distractions?*

- *How do higher education students vary in their attentional states and use of attention-regulation strategies in an emergent distance learning context?*
- *To what extent does higher education students’ personality relate to their profile membership for attentional state and the use of attention-regulation strategies in an emergent distance learning context?*

Our specific hypotheses therefore are:

- H1: Distance learning profile membership groups, as derived from the meta-attention framework, will exhibit significant differences in time spent on the Internet and social media outside of studying.
- H2: Younger students who spend more daily time on the Internet and social media outside of academic purposes will show greater susceptibility to distractions and challenges to their attentional states during distance learning.
- H3: Personality traits will significantly influence students' attentional states and regulatory strategies. Specifically:
  - H3a: Conscientiousness and agreeableness will be positively associated with clusters exhibiting better attentional states and regulation strategies during distance learning.
  - H3b: Extraversion, openness, and neuroticism will be positively linked to clusters prone to social media distractions and weaker regulation skills during distance learning.

## Method

### Procedure

Universities in Austria moved towards distance learning in March 2020 due to the outspread of COVID-19. In April 2020, students were recruited to take part in the study via their university lecturers. A convenience sampling strategy [38] was used, in accordance with the specific courses offered by the relevant faculties (teacher training, education, psychology, sociology, informatics, philosophy, and others). University teachers were invited to participate in the study and forward the link to students in one of their courses. As a result, the response rate cannot be calculated, and the data are not representative. Students who were invited to participate were asked to complete the study questionnaire; they were provided with information about the study and asked to actively tick an informed consent box before they could begin. As no minors took part in the survey, ethics approval was not required.

### Compliance with ethical standards

All participants gave informed consent. Participants were provided with detailed information about the study and asked to actively tick an informed consent box before they could begin. The study was conducted in accordance with the Declaration of Helsinki. According to national regulations (see Federal Ministry Republic of Austria) as well as regulations of the University of Vienna (University of Vienna Ethics Committee) ethics approval is not mandatory in non-clinical studies if no minors take part in the survey. The authors have no conflicts of interest to declare.

### Participants

The study included 538 undergraduate higher education students; however, data from 138 participants were excluded due to missing data. The final sample consisted of 400 students, with 309 females and 91 males. The mean age of the sample was 25.9 years (min = 18, max = 81, *SD* = 8.8). The participants studied humanities or social sciences disciplines: 45.8% teacher education, 13.8% educational science, 6% psychology, 6% anthropology, and 28.4% other disciplines.

### Instruments and their validation

The *amount of daily internet use* was assessed with the following questions: “*How many hours do you spend online per day?*” and “*How many hours do you spend online outside of study per day*?” Participants were asked to refer to the time before (retrospective) and during (in situ) the COVID-19 lockdown when answering these two questions.

*Personality traits* were measured using the short version of the Ostendorf [39] MRS-inventory as adapted by Schallberger and Venetz [40]. This questionnaire measures five dimensions of personality (agreeableness, conscientiousness, extraversion, neuroticism, openness). Participants responded on a 6-point Likert scale. The Cronbach’s alphas were ranging between α = 0.64 and 0.87.

*Online learning attention and regulation* were assessed using an adapted version of the Online Learning Motivated Attention and Regulatory Strategies (OL-MARS) scale [4]. The original scale consists of 19 items that assess two dimensions: attentional knowledge and regulation of attention. Responses are rated on a 5-point Likert scale. We adapted the scale to make it applicable to higher education students in the context of emergent distance learning. Perceived attention discontinuity (PAD) comprised four items (e.g., *During my online course, I visit websites or applications that are irrelevant to my learning*.; *⍺* = 0.74), awareness of social media notifications (SMN) comprised three items (e.g., *When I see, hear or feel signals, sounds or vibrations from my smartphone, I check them immediately*.; *⍺* = 0.77), and mental and behavioral regulation strategies (RS) comprised four items (e.g., *I call on myself to first complete the tasks at hand before using the Internet for other purposes.*; *⍺* = 0.52). Removing one behavioral regulation item (i.e., “When I study, I log off my Facebook account or close the instant messaging software so I can focus on my work.”) improved the RS subscale’s internal consistency (from α = 0.52 to α = 0.65). However, this item, addressing social media use, was retained due to its established relevance in predicting behavioral regulation [4, 41].

### Data analysis

For the person-oriented approach, a cluster analysis was performed to identify students’ different knowledge and regulation of attention profiles. Based on Audigier, Husson, and Josse [42], we conducted hierarchical clustering on principal components (HCPC) analysis, followed by k-means clustering, using the FactoMineR and FactoShiny packages in R. The standardized data were examined using hierarchical cluster analysis (with Ward’s linkage algorithm based on squared Euclidean distances as the distancing metric) to minimize intra-group variance [43, 44].

To examine the stability and reliability of the cluster solution, we followed a sequence of steps to ensure that the solution would retain the same meaning in independent samples. First, all values were transformed into standardized z-scores. Second, using cross-validation, the sample was randomly split into two datasets: 70% of the data was used as a training dataset, and the remaining 30% was used as a test dataset. These datasets were cluster-analyzed separately before the full analysis [45]. The HCPC and *k*-means procedures were performed separately on the two datasets and led to two sets of cluster solutions. To evaluate the quality of the clusters using existing internal validity criteria, a Silhouette analysis was performed. To examine the predictive validity of the analyses, we first checked whether the independent datasets had the same number of clusters. If the number of clusters was identical, we built a classification rule using predictive discriminant analysis techniques, based on the cluster solution obtained in the training dataset. Next, we used the classification rule to assign the test dataset cases into groups [46]. A linear classification rule in R’s Mass package was used to conduct a discriminant analysis among clusters to verify the cluster solution and assess classification adequacy. Hit rates of at least 75% suggest cluster solution stability and reliability [47]. Once we established the best clustering result, the final stage was to feed all of the data samples, composed of both the training and testing datasets, into the best clustering algorithm, in order to obtain the “best” student groups.

Finally, by using a variable-oriented approach, we checked for associations between the five-factor personality traits, demographic data, internet use, and the knowledge and regulation of attention components. As stated in Assuah et al. [48], the categorical variables, or the probability of assigning category membership to a dependent variable can be predicted using multinomial logistic regression, like with its binary counterpart, which assesses the likelihood of belonging to a category by maximum likelihood estimation. We used this model to describe the conditional probability of a student’s personality trait with respect to a multinomial discrete choice (i.e., attention and regulation strategies clusters). After the multinomial regression model was constructed, its parameters were utilized to make predictions about how likely each occurrence was relative to the reference category [49]. Consequently, to determine a student’s cluster membership by their personality traits, a multinomial logistic regression was employed using the brglm2 package in R.

## Results

### Assumption checking, descriptive statistics, and correlation matrix for the study variables

Before conducting the analyses, relevant assumptions were checked for both the one-way ANOVA and the multinomial logistic regression. Independence of observations was ensured through the study design. Levene’s test was nonsignificant (*p* > 0.05), confirming that the assumption of homogeneity of variances was met for the one-way ANOVA. Furthermore, skewness and kurtosis values for the cluster-analysis variables remained within the acceptable ± 3 range, indicating no substantial violations of normality.

For the multinomial logistic regression, Multicollinearity was assessed using the Variance Inflation Factor (VIF), with all predictors exhibiting VIF values below 3, indicating acceptable levels of collinearity. The Box-Tidwell approach revealed no significant deviations from linearity (*p* > 0.05), and the sample size was sufficient for each predictor category. Consequently, all assumptions were met, and the multinomial logistic regression was carried out without further modifications.

Descriptive statistics and the correlation matrix for the study variables are summarized in Table 1. Notably, neuroticism showed high positive correlations with online time spent outside studying before emergent distance learning, PAD, and SMN, and high negative associations with RS. Conscientiousness had a high negative correlation with online time spent outside studying during emergent distance learning and with the same OL-MARS variables; however, it had a high positive correlation with RS. Openness and extraversion had significant negative relationships with online time spent outside studying before emergent distance learning and PAD, while openness had a significant negative relationship with SMN. Furthermore, students’ age was negatively correlated with neuroticism, SMN, and RS, and positively correlated with extraversion.

**Table 1:** Mean, standard deviation, and intercorrelations among study variables

	Study variables	M (*SD*)	1	2	3	4	5	6	7	8	9	10	11	12	13
1	Age	25.92 (8.80)													
2	Gender	.077 (041)	-.11*												
3	Online study time before DL	2.66 (1.71)	.02	.02											
4	Online free time before DL	2.88 (1.70)	-.08	-.08	.02										
5	Online study time during DL	4.93 (2.20)	-.09	.10*	.56**	-.11*									
6	Online free time during DL	3.58 (2.34)	-.03	-.05	-.09	.64**	-.23**								
7	Openness	4.54 (0.97)	.01	.12*	.03	-.11*	.08	-.09							
8	Extraversion	4.31 (1.17)	.13**	.11*	.03	-.20**	.04	-.09	.22**						
9	Conscientiousness	4.74 (0.94)	.05	.17**	.05	-.19**	.08	-.13**	.04	.15**					
10	Neuroticism	3.20 (0.89)	-.13**	.16**	.05	.16**	.04	.08	-.12*	-.38**	-.01				
11	Agreeableness	2.17 (0.67)	-.05	-.03	.07	.08	-.02	.03	-.21**	-.18**	-.15**	.23**			
12	PAD	2.21 (0.91)	-.07	-.06	.05	.18**	-.07	.20**	-.14**	-.17**	-.22**	.18**	.05		
13	SMN	3.18 (1.06)	-.20**	.16**	.04	.09	-.03	.06	-.14**	-.04	-.14**	.15**	-.01	.38**	
14	RS	3.52 (0.75)	-.12**	.12*	.06	-.08	.09	-.04	.08	-.01	.15**	-.16**	.02	-.02	-.11*

*M *Mean, *SD *Standard deviation, *DL *Distance learning, *PAD *Perceived attention discontinuity, *SMN *Social media notification, *RS *Mental and behavioral regulation strategies

*p < .05

**p < .01

### Clusters describing attention and regulation strategies

Two cluster analysis methods were conducted on the training dataset: the HCPC and *k*-means techniques produced three clusters. If a cluster solution has good quality, the elements within a cluster should be cohesive; nevertheless, each cluster should be quite different from the other clusters [43, 50]. The Silhouette coefficient was .30 for the training dataset, indicating a fair cluster quality. After conducting HCPC and *k*-means cluster analysis for the test dataset following the same procedure, a three-cluster solution was also identified with a Silhouette coefficient of .30. The number of clusters generated in the two separate independent samples was the same, indicating that we could proceed to examine whether the clusters in the two datasets carried the same meaning [47].

A predictive discriminant analysis was conducted on the training dataset to develop a linear classification rule. The cluster solution generated by the training dataset was the dependent variable and the three dimensions measuring attention were the independent variables. The classification rule was then used to “predict” the group membership of the cases in the test dataset. Stable cluster patterns across independent samples indicated that the classification rule developed in the training dataset would replicate the cluster patterns found in the test dataset. The agreement, measured by hit rates, between the “predicted” membership and the cluster solution in the test dataset is displayed in Table 2. The rows in Table 2 correspond to the cluster assignment generated in the training and test datasets and the columns in Table 2 correspond to the cluster solution when cases were assigned using the classification rule. The “hit rates” are displayed along the diagonal of Table 2 and show the percentage of cases within each cluster that were accurately classified by the classification rule generated from the training dataset. On average, the model had good accuracy: around 97.4% of the cases had their cluster membership accurately predicted by the rule obtained from the training dataset. This high agreement provided evidence for the external validity of the cluster patterns across independent samples. Through this process, where the cluster size was found to be stable, the original cluster solution was confirmed. This result showed a stable cluster solution with fewer than 10% of observations being assigned to a different group.

**Table 2:** Hit rates for replication in test dataset (n2 = 116) using a classification rule generated in training dataset (n1 = 284)

Cluster datasets		Cluster 1	Cluster 2	Cluster 3	Total
***Cluster solution in training dataset***					
Cluster 1	N	113	5	3	121
	% within Cluster	93.38%	4.13%	2.47%	100%
Cluster 2	N	0	93	0	93
	% within Cluster	0%	100%	0%	100%
Cluster 3	N	0	0	70	70
	% within Cluster	0%	0%	100%	100%
***Cluster solution in test dataset***					
Cluster 1	N	47	1	0	48
	% within Cluster	97.9%	2.08%	0%	100%
Cluster 2	N	0	38	0	38
	% within Cluster	0%	100%	0%	100%
Cluster 3	N	0	0	30	30
	% within Cluster	0%	0%	100%	100%

**Fig. 1:**
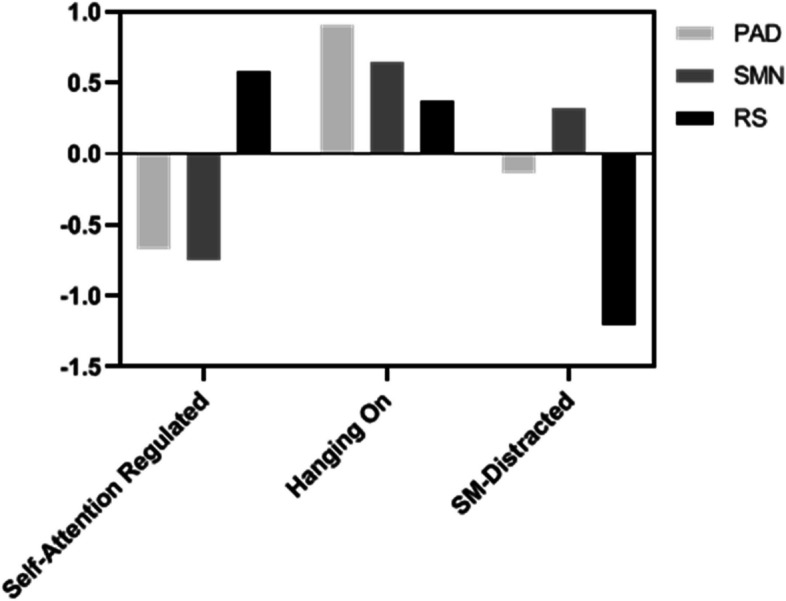
Mean values of clustering variables across clusters’ profiles

The HCPC and *k*-means analyses of the dataset identified three distinct clusters, aligning with the meta-attention framework, which incorporates components of perceived attention discontinuity (PAD), social media notifications (SMN), and regulation strategies (RS). Cluster 1: Self-Attention Regulated (*n* = 160, 40%, 38 males, 122 females): students with low PAD and SMN scores, and high RS scores, who reported the least social media notification distraction and moderate attention issues. Cluster 2: Hanging-On (*n* = 105, 26%, 30 males, 75 females): students with medium RS scores and high PAD and SMN scores, who reported having a clear perception of their attention state and utilized some strategies to regulate their attention in distance learning; these students were trying to catch-up. Cluster 3: Social Media Distracted (*n* = 135, 34%, 23 males, 112 females): students in this cluster scored lowest on RS, with low PAD scores compared to the Self-Attention Regulated cluster and high SMN scores compared to the Hanging-On cluster; students in Cluster 3 were aware of their social media attention problems but had poor strategies to regulate them. Figure 1 shows the distribution of Mean values of clustering variables across clusters’ profiles. A follow-up MANOVA was conducted to assess the degree of difference among the three clusters, not to validate them, as clustering is exploratory and identifies patterns rather than testing hypotheses [51]. Nevertheless, MANOVA is effective for examining inter-cluster differences across indicators, as used in prior studies on attention and regulation strategies [4, 52]. Results indicated significant differences among the three clusters across the variables, as shown in Table 3. A Bonferroni post hoc analysis confirmed that all pairwise comparisons were significant (*p* < 0.05), except for mental and behavioral regulatory strategies use between the Self-Attention Regulated and Hanging-on clusters (*p* = 0.19).

**Table 3:** Descriptive statistics and MANOVA results of clustering variables across clusters' profiles

Clusters' profiles	Cluster 1 (*n*= 160) M(SD)	Cluster 2 (*n*=105) M(SD)	Cluster 3 (*n*=135) M(SD)	F
	*Self-* *Attention Regulated*	*Hanging-on*	*SM-Distracted*	
Perceived attention discontinuity (PAD)	-0.67 (0.55)	0.91 (0.83)	-0.13 (0.83)	168.90^**^
Social media notification (SMN)	-0.74 (0.81)	0.64 (0.74)	0.31 (0.80)	123.78^**^
Mental and behavioral Regulatory Strategies (RS)	0.58 (0.69)	0.37 (0.62)	-1.21 (0.76)	220.66^**^

**p < .01; *M* Mean, *SD *Standard deviation, *SM-Distracted* Social media-Distracted

In the next stage of the analysis, we examined the clusters in terms of gender, age, and internet usage. Cluster membership was associated with gender (*X*^*2*^ = 15.06, *df* = 2,* p* < 0.005, Cramer’s V = 0.19). Significant differences between cluster groups were found for mean age (*M* = 25.29; *SD* = 8.08; *F* = 3.19, *df* = 2, *p* = 0.009). Bonferroni post hoc multiple comparison tests showed no difference in age between the Self-Attention Regulated (*M* = 27.04, *SD* = 10.8) and Hanging-On (*M* = 25.95, *SD* = 6.81) clusters; and no difference in age between the Hanging-On and Social Media-Distracted clusters. However, participants in the Self-Attention Regulated cluster were older than those in the Social Media-Distracted cluster (*M* = 24.17, *SD* = 5.72).

**Fig. 2:**
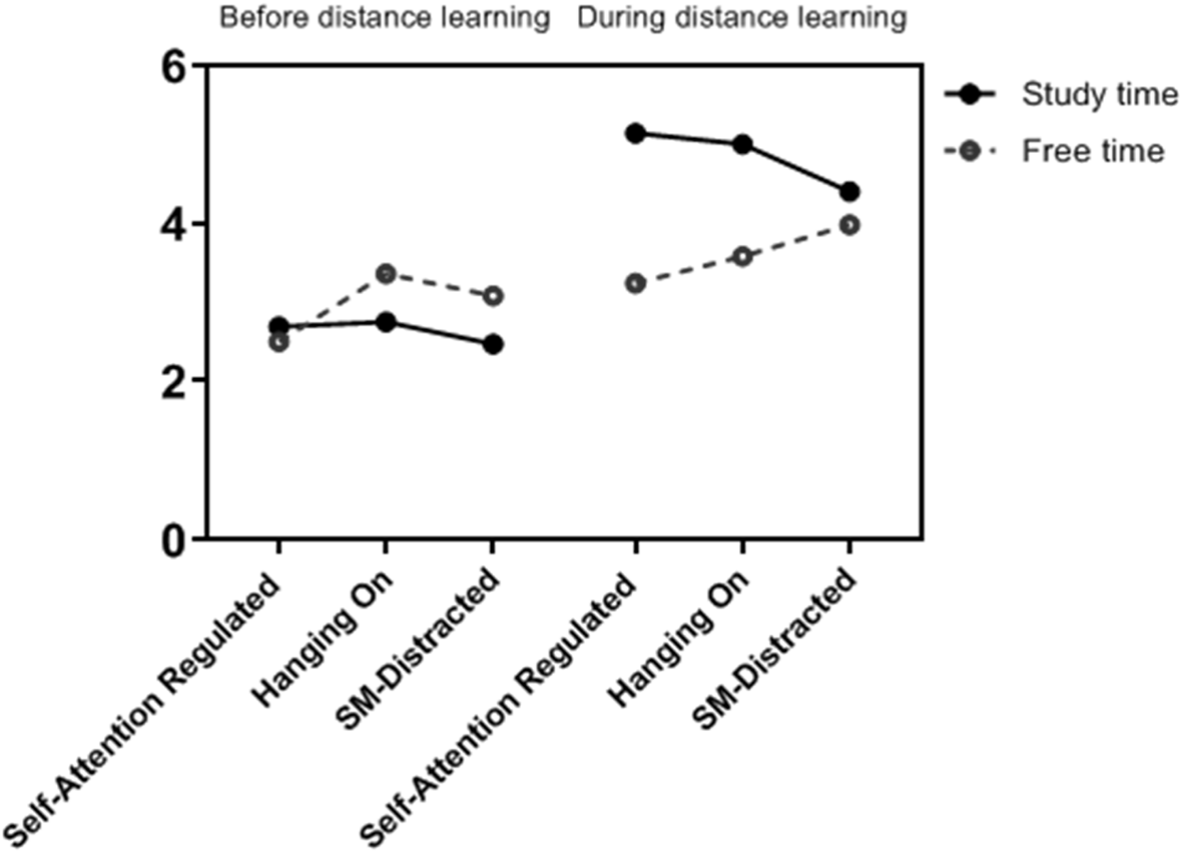
Mean values of daily hours of internet use-related validation variables across clusters

We also examined the differences between clusters for using the Internet for study and outside of study before and during emergent distance learning. As shown in Fig. 2, and Table 4 there was a statistically significant difference between the cluster groups on the combined internet use variables. While MANOVAs demonstrated no significant differences between cluster groups in mean online study time before COVID-19 (*F* = 0.968, *df* = 2, *p* > 0.05), there was a significant difference between cluster groups in mean online study time during distance learning (*F* = 3.86, *df* = 2, *p* < 0.001). Specifically, Bonferroni post hoc multiple comparison tests showed that Self-Attention Regulated students reported engaging in significantly more online study time during distance learning than Social Media-Distracted students.

**Table 4:** MANOVA and Bonferroni results for age and internet use variables for the three-cluster solution

Cluster comparison variables	Cluster 1 Self-Attention Regulated	Cluster 2 Hanging-On	Cluster 3 SM-Distracted	F	Bonferroni post hoc multiple comparison tests
	*M(SD)*	*M(SD)*	*M(SD)*		
Age	27.04 (10.8)	25.95(6.81)	24.17(5.72)	4.86**	Cluster 1 reported significantly higher scores than Cluster 3.
Online study time before distance learning	2.69 (1.90)	2.75 (1.54)	2.47 (1.47)	.968	No Significant differences between the three clusters.
Online free time before distance learning	2.50 (1.44)	3.36 (1.83)	3.08 (1.87)	10.34**	- Cluster 2 and Cluster 3 reported significantly higher time than Cluster 1. - No Significant differences between Cluster 2 and Cluster 3.
Online time for study during distance learning	5.14 (2.24)	5 (2.28)	4.40 (2.07)	3.866*	- Cluster 1 reported significantly higher time than Cluster 3 - No Significant differences between Cluster 2 and Cluster 3. - No Significant differences between Cluster 1 and Cluster 2.
Online free time during distance learning	3.24 (2.11)	3.58 (2.34)	3.98 (2.65)	4.011*	- Cluster3 reported significantly higher time than Cluster 1 - No Significant differences between Cluster 2 and Cluster 3. - No Significant differences between Cluster 1 and Cluster 2.

*p < .05; **p < .01; *M* Mean; *SD* Standard deviation

Regarding free time before and during distance learning, there were significant differences between cluster groups in mean online free time before and during distance learning (*F* = 10.34, *df* = 2, *p* = 0.01, *F* = 4.011, *df* = 2, *p* = 0.04, respectively). Bonferroni post hoc analyses showed that before distance learning Hanging-On and Social Media-Distracted students engaged in significantly higher daily time on activities outside of studying than did Self-Attention Regulated students. This significant aspect remained the same for the students in the Social Media-Distracted cluster, where their daily time on activities outside of studying was significantly higher than for Self-Attention Regulated students.

### The influence of personality traits on attention and regulation strategies

To investigate to what extent personality traits predicted cluster membership, we first conducted a descriptive analysis of each personality trait in each cluster to uncover students’ personality trait levels (see Table 5).

**Table 5:** Personality trait descriptive analysis in each cluster; values represent means (M) and standard deviations (SD)

Personality trait	Self-Attention Regulated	Hanging -on	SM-distracted
	*M(SD)*	*M(SD)*	*M(SD)*
Agreeableness	2.15 (0.70)	2.13 (0.76)	2.21(0.83)
Extraversion	4.35 (1.08)	4.37 (1.18)	4.19(1.26)
Conscientiousness	4.93 (0.86)	4.58 (1.05)	4.63(0.88)
Openness	4.73 (0.91)	4.37(1.02)	4.43(0.96)
Neuroticism	3.12 (0.86)	3.03 (0.85)	3.43 (0.9)

Secondly, to identify to what extent each personality trait played a significant role in determining their cluster profile membership, we conducted a multinomial logistic regression with Clusters 1–3 as outcomes and the five personality traits as predictors. The model predicting cluster membership was significant (*χ2*(10) = 39.62, *p* < 0.001, Nagelkerke *R2* = 0.121). This model with five predictors also explained approximately 12% of the variance associated with students’ membership in each of the three clusters.

Table 6 shows the parameter estimates; this also provides the raw score coefficients (adjusted for the presence of the other predictors in the model) associated with each of the predictors (see Column β). The partial regression coefficients were tested for statistical significance using the Wald test. The odds ratio, which is the primary part of the output, is shown as Exp (β). Overall, openness, conscientiousness, and neuroticism, but not agreeableness and extraversion, significantly predicted cluster membership.

**Table 6:** Multinomial logistic regression results predicting students’ distance learning attention and regulation strategies profiles membership

Students’ attention and regulation strategies profiles	Personality traits	Β	Wald	Exp (β)
Self-Attention Regulated Vs. Hanging-on	Intercept	−4.254	10.123**	
	Extraversion	-.093	.557	.911
	Agreeableness	.207	1.280	1.230
	Conscientiousness	.467	10.299**	1.596
	Openness	.462	10.674**	1.587
	Neuroticism	.100	.357	1.105
Self-Attention Regulated Vs. SM-Distracted	Intercept	−1.750	2.092	
	Extraversion	-.088	.600	.916
	Agreeableness	.152	.846	1.165
	Conscientiousness	.399	8.535**	1.491
	Openness	.325	5.933*	1.384
	Neuroticism	-.439-	8.426**	.644
SM-Distracted Vs. Hanging-On	Intercept	−2.504	3.686	
	Extraversion	-.005	.002	.995
	Agreeableness	.055	.088	1.056
	Conscientiousness	.068	.233	1.070
	Openness	.137	.966	1.147
	Neuroticism	.539	10.082**	1.714

^*^p < .05; **p < .01

The first line shows contrasting linear equations. The raw score coefficients associated with conscientiousness and openness positively and significantly predicted students’ membership in the Self-Attention Regulated cluster, in contrast to the Hanging-On and Social Media-Distracted clusters. The odds ratio, adjusted for the other predictor variables in the model, yielded an interpretation of the dynamics of the predictor variables. For example, the conscientiousness trait was associated with an adjusted odds ratio of 1.59. This means that a unit increase in conscientiousness increases the odds of a student being in the Self-Attention Regulated cluster by 1.59 versus the odds of being in the linear equations cluster membership, controlling for the other predictors. In the same vein, it was shown that a unit increase in openness increases the odds of a student being in the Self-Attention Regulated cluster by 1.58 versus the odds of being in the linear equations cluster membership, controlling for the other predictors.

On the other hand, only the neuroticism trait positively and significantly predicted students’ membership in the Social Media-Distracted cluster in contrast to the Self-Attention Regulated and Hanging-On clusters. Neuroticism was associated with an adjusted odds ratio of 1.71. This means that a unit increase in neuroticism increases the odds of a student being in the Social Media-Distracted cluster versus the odds of them being in the linear equations cluster membership, controlling for the other predictors.

## Discussion

Using a cluster analysis approach, this study first explored how the sudden shift from face-to-face learning to emergent distance learning influenced students’ attentional state and regulation strategies. Specifically, the students’ distance-learning attention and regulation profiles were identified. Based on the variable-oriented approach, the influence of the Big Five Personality traits and students’ demographic characteristics on their distance-learning attention and regulation profiles was examined, and the purpose and intensity of the time spent online before and during the COVID-19 pandemic were compared across these profiles.

The cluster analysis results revealed heterogeneity in students’ attention and regulation strategies during distance learning. These findings align with the meta-attention framework and corroborate the conclusions drawn by Wu [4]. In support of our aims, we identified three distinct distance-learning attention and regulation strategies clusters: (1) the Self-Attention Regulated cluster, (2) the Hanging-On cluster, and (3) the Social Media-Distracted cluster. It is of some concern that the Hanging-On (26%) and Social Media-Distracted (34%) clusters indicated that, in general, students experienced more difficulty in managing their response to distractions during emergent distance learning and regulating their attention and efforts during distance education. Using a person-specific technique (HCPC followed by *k*-means analysis), our Self-Attention Regulated and Hanging-On clusters were similar to the two clusters reported by Wu [4]. Accordingly, the author reported that Self-Attention Regulated students had fewer attention problems, spent less time on social media daily and per visit, and made fewer average visits per day compared to Hanging-On students, who had more attention problems and spent more time on social media daily and per visit, and made more average visits per day [4]. However, in contrast to Wu [4], we identified the Social Media-Distracted cluster, whose members had low perceived attention discontinuity scores, high social media notification scores, and the poorest attention regulation. This cluster (i.e., Social Media-Distracted) underscores the crucial role of working memory and attention in managing multiple tasks. During the COVID-19 pandemic, constant access to digital devices enabled students to engage in online courses while being distracted by social media notifications, placing heavy demands on working memory and dividing attention. As cognitive resources are limited, this interference impairs task performance [53]. Our findings, consistent with prior studies [4, 47, 54], reveal that students spend an average of nearly five hours daily on their phones, with heavy users showing near-constant engagement. This pervasive device use, driven by the constant urge to check social media due to the expectation of notifications and curiosity about ongoing developments during the pandemic, highlights the need for effective attention regulation.

Profiling the clusters by the Big Five Personality traits revealed a significant effect between personality and the students’ identified distance-learning attention and regulation profiles. More specifically, conscientiousness, openness, and neuroticism have predictive effects on students’ distance-learning attention and regulation strategies profile membership.

Consequently, students’ attentional state and regulation strategies for distance learning are influenced by their personality traits, and these strategies change as they become older. Compared to the Self-Attention Regulated cluster, our results showed that the Hanging-On and Social Media-Distracted clusters were younger. This may be because older students may have been able to better regulate their attention during distance learning and they remained unaffected by distractions because of this greater self-attention regulation than younger students [21, 55].

Another important finding was that students in the Self-Attention Regulated group exhibited significantly higher levels of conscientiousness and openness. According to the meta-attention framework, these students experienced minimal perceived attention discontinuity, were less distracted by social media notifications, employed robust mental and behavioral regulatory strategies, spent less time on non-academic activities before and during the pandemic, and devoted more time to studying during the pandemic. Individuals with high conscientiousness often prioritize fulfilling their tasks and obligations, which explains the reported negative relationship between conscientiousness and social media usage [30, 56]. Our results also support studies by Ross et al. [57] and Andrews et al. [26], which suggest that individuals high in conscientiousness may limit their use of social media to avoid procrastination and distractions from work.

Interestingly, we found that students in the Self-Attention Regulated cluster possessed high openness. This is inconsistent with earlier research that found that openness is positively associated with greater use of social media systems such as Facebook (e.g., [57, 58]), but is consistent with Vaid and Harari [59], who found a negative relationship between social media use and openness. One possible explanation for our results might be that students with higher openness tend to be more curious and thus concentrate more on distance learning in emerging situations such as the pandemic, which could explain why they get less distracted than if they were studying in traditional face-to-face learning contexts.

On the other hand, neuroticism is seen as crucial in determining whether or not a student will fall into the Social Media-Distracted cluster. Students in this group tended to be younger than 25 years old, had lower attentional discontinuity and regulatory strategies scores, were more distracted by social media notifications, and spent more time on the internet and social media outside of studying both before and after the epidemic. One of the earliest arguments against internet use by neurotic individuals is that it is inherently dangerous due to the presence of threats and weaknesses in publicly accessible networks [60]. The COVID-19 pandemic, however, has many elements that make it exceptionally stressful for students. Stress and anxiety are elicited by physical isolation or social distancing as well as complete disruption to daily routines. Our results are consistent with recent research that suggests that persons with high neuroticism utilize social media frequently, especially to prevent loneliness [1, 56].

Although our study originated from emergency distance learning conditions [61], the transition to digitally mediated learning persists [62, 63]. Our findings emphasize that attentional regulatory strategies remain essential, as devices and online platforms continue to be integral in education. Structured course designs, as suggested by Jaggars and Xu [64], can reduce multitasking by simplifying content and providing clear guidelines. Multimedia learning principles [65] further highlight the value of curated content to alleviate cognitive overload. Finally, personalized approaches, such as those discussed by Ma et al. [37], show promise, with conscientious students benefiting from flexible structures and those high in openness needing targeted strategies to manage distractions.

## Conclusion, limitations, and future research

This study provides valuable insights into how personality traits factored into higher education students’ attention and regulatory strategies during distance learning in the context of the initial COVID-19 lockdown. This study demonstrated that students high in conscientiousness and openness were more likely to regulate their own attention and use effective attention and regulation strategies, while those with high neuroticism are more likely to be distracted by social media and have attention problems. Additionally, we found that younger students especially are more prone to social media distractions.

A strength and a limitation of the current study is that it was conducted during the initial lockdown of the COVID-19 pandemic. On the one hand, this was a special situation that required special attention regulation in students. On the other hand, the results have limited generalizability. A methodological problem is that we used cross-sectional data and self-reports. Future studies may explore the research questions in a way that uses other data sources, such as exam grades. Moreover, we used the Facebook channel as an example of social media in this study. However, students also use other social media platforms, such as TikTok. We believe that this partly explains the lower internal consistency scores observed for the RS subscale. Finally, the sample consisted mostly of female participants. Therefore, future research should also include more male, as well as non-binary participants to obtain a more diverse sample.

## Data Availability

The data that support the findings of this study are available on request from the first author.

## Abbreviations

OL-MARS: Online learning motivated attention and regulation
PAD: Perceived attention discontinuity
SMN: Social Media Notifications
RS: Mental and behavioral regulation strategies
SM-Distracted: Social Media-Distracted
DL: Distance Learning
VIF: Variance Inflation Factor
M: Mean
SD: Standard deviation
